# Spotting *Culex pipiens* from satellite: modeling habitat suitability in central Italy using Sentinel-2 and deep learning techniques

**DOI:** 10.3389/fvets.2024.1383320

**Published:** 2024-07-04

**Authors:** Carla Ippoliti, Lorenzo Bonicelli, Matteo De Ascentis, Susanna Tora, Alessio Di Lorenzo, Silvio Gerardo d’Alessio, Angelo Porrello, Americo Bonanni, Daniela Cioci, Maria Goffredo, Simone Calderara, Annamaria Conte

**Affiliations:** ^1^Istituto Zooprofilattico Sperimentale dell’Abruzzo e del Molise “G. Caporale”, Teramo, Italy; ^2^Department of Engineering “Enzo Ferrari”, University of Modena and Reggio Emilia, Modena, Italy

**Keywords:** *Culex pipiens*, vector-borne diseases, satellite Earth Observation, Copernicus Sentinel-2, deep learning, convolutional neural network, West Nile virus, Italy

## Abstract

*Culex pipiens*, an important vector of many vector borne diseases, is a species capable to feeding on a wide variety of hosts and adapting to different environments. To predict the potential distribution of *Cx. pipiens* in central Italy, this study integrated presence/absence data from a four-year entomological survey (2019–2022) carried out in the Abruzzo and Molise regions, with a datacube of spectral bands acquired by Sentinel-2 satellites, as patches of 224 × 224 pixels of 20 meters spatial resolution around each site and for each satellite revisit time. We investigated three scenarios: the baseline model, which considers the environmental conditions at the time of collection; the multitemporal model, focusing on conditions in the 2 months preceding the collection; and the MultiAdjacency Graph Attention Network (MAGAT) model, which accounts for similarities in temperature and nearby sites using a graph architecture. For the baseline scenario, a deep convolutional neural network (DCNN) analyzed a single multi-band Sentinel-2 image. The DCNN in the multitemporal model extracted temporal patterns from a sequence of 10 multispectral images; the MAGAT model incorporated spatial and climatic relationships among sites through a graph neural network aggregation method. For all models, we also evaluated temporal lags between the multi-band Earth Observation datacube date of acquisition and the mosquito collection, from 0 to 50 days. The study encompassed a total of 2,555 entomological collections, and 108,064 images (patches) at 20 meters spatial resolution. The baseline model achieved an F1 score higher than 75.8% for any temporal lag, which increased up to 81.4% with the multitemporal model. The MAGAT model recorded the highest F1 score of 80.9%. The study confirms the widespread presence of *Cx. pipiens* throughout the majority of the surveyed area. Utilizing only Sentinel-2 spectral bands, the models effectively capture early in advance the temporal patterns of the mosquito population, offering valuable insights for directing surveillance activities during the vector season. The methodology developed in this study can be scaled up to the national territory and extended to other vectors, in order to support the Ministry of Health in the surveillance and control strategies for the vectors and the diseases they transmit.

## 1 Introduction

Vector-borne diseases (VBDs), a category of zoonosis, are transmitted to humans and animals (primarily ruminants) through vectors including mosquitoes, ticks, and fleas. Recently, VBDs have emerged as a significant threat to human health in temperate areas (1). In Eastern, Western and Southern Europe, including Italy, the West Nile virus (WNV) is the most widespread mosquito-borne zoonosis (2). In its transmission cycle, birds act as primary and amplifying hosts, with mosquitoes transmitting the virus to other birds; humans and other mammals are considered dead-end hosts, as they generally do not contribute to virus transmission, often remaining asymptomatic (3).

In Europe, the *Culex pipiens* [from now on *Cx. pipiens*, (4)] is recognized as the main WNV vector (5, 6). The presence of *Cx. pipiens* in the world’s temperate climatic regions and its ability to transmit zoonotic pathogens, other than WNV, such as Rift Valley fever (7), Usutu and Japanese encephalitis (8), made *Cx. pipiens* one of the most important mosquito species regarding public health. *Culex pipiens* is a species able to feed on birds and mammals, including humans (9, 10), and it breeds in a wide variety of environments, either in rural and urban cycles, tolerating also human-altered ones (11).

This ability to adapt to a wide range of environments rely on its characteristics to tolerate a number of conditions, including the adaptability to many hosts for feeding, the flexibility to laying eggs in presence of water ponds or humid soil, and the environmental tolerance for resting and breeding (11). This complexity contributes to the species characteristics in terms of trophic behavior and vectorial capacities. A fine understanding of the *Cx. pipiens*’ habitat suitability that facilitates survival, reproduction and dispersal becomes of paramount importance for determining the risk of local establishment, persistence and spread, developing efficient (i.e., species-, place- and time specific) vector monitoring.

In Italy, *Cx. pipiens* is the species most frequently found to carry WN virus (5, 12). Across the country, *Cx. pipiens* population is characterized by seasonal dynamics across the year, with its maximum population abundance during summer (13, 14). The intensity and inter-annual variations of vector populations have been frequently associated to temperature, rainfall, humidity, vegetation, i.e., to climatic-environmental drivers. Temperature has been most frequently reported as the most influential variable affecting mosquito population dynamics (15–17). Temperature drives the vector competence, by accelerating the virus replication within the insects, prolonging their breeding season (18, 19), increasing mosquito abundance (20) and their infection rate (21). Rainfall has been found to play a significant role in many studies (19, 22), although its impact remains a topic still controversial in literature (23). On one hand, rainfall creates pools of water, which serve as suitable breeding sites for mosquitoes, thereby increasing species abundance. On the other hand, excessive rainfall can damage larval habitats flushing aquatic environments. Additionally, air humidity in preceding months is another factor associated with the abundance of mosquitoes (13). The length of daylight was another abiotic factor associated with *Cx. pipiens* population growth in Italy (22, 23). Vegetation and vegetation indices are other parameters correlated with the vectors’ behavior and their biological cycle (23, 24), although not always resulted relevant (19): the presence and density of green biomass provide sugar feeding supplies for adult mosquitoes, potential resting and protection from climatic conditions. A combination of these (and other) drivers have been used to classify Italian territory into different ecoregions (25).

In species distribution modeling studies aiming to associate spatial environmental characteristics with the presence/abundance of vectors, the Random Forest machine learning method is probably the most commonly used approach (26–28) among others, alongside with MaxEnt (29, 30). For an in-depth description of Machine Learning (ML) based species distribution modeling, a comprehensive review is available in Zhang and Li (31). In another research, three supervised learning models, k-nearest neighbor (kNN), artificial neural network (ANN), and support vector machine (SVM) were used to predict mosquito abundance based on socioeconomic and landscape patterns (32). Other authors used ML methods to predict West Nile virus outbreaks or WNV infection rates in *Culex* mosquitoes with eco-climatic drivers (33, 34).

The most common statistical methodologies focused on epidemiological modeling of main VBDs in Europe rely on pre-calculated indices and factors, such as NDVI (Normalized Difference Vegetation Index), presence of standing water indicated by NDWI (Normalized Difference Water Index) or soil moisture levels (Moisture Index) (24, 35). Those indices are well from derived from Earth Observation (EO) data: EO data has played a crucial role in the study of VBDs, particularly in the realm of epidemiological modeling and in understanding the environmental factors influencing disease transmission dynamics. EO data, collected by sensors onboard satellites, provides data about Earth’s surface across a number of wavelengths. This data provides measurements of surface temperature, chlorophyll presence, water presence, soil characteristics, land cover, among others relevant features of Earth surface supporting mosquito populations life cycle, hence possibility of VBDs transmission (36). The frequency, consistency and regularity of data acquisition generates continuous datasets able to depict environmental features crucial for understanding mosquito habitats, either as larval sites, breeding or resting places. By analyzing satellite data alongside epidemiological information on diseases, environmental risk factors associated with VBDs can be identified, assisting in pinpointing areas with high vector concentrations or conducive environmental conditions for disease transmission (24). In turn, these results enhance understanding of disease transmission mechanisms and interactions between the environment and human health, thereby facilitating the development of more effective prevention and control strategies.

To date, new large EO datasets are available at unprecedented spatial, spectral and temporal resolutions, as those produced by the European Copernicus program. The satellite data of the Sentinel-2 mission, in particular, are dedicated to land and vegetation monitoring, and through the Multi-Spectral Instrument (MSI) carried onboard, they return an optical multispectral “photograph” of the territory they fly over every 5 days. The Sentinel-2 constellation comprises Sentinel-2A (in orbit since June 23, 2015) and Sentinel-2B (in orbit since March 7, 2017) satellites, orbiting the Earth simultaneously on the same sun-synchronous orbit, offset by 180 degrees. The MSI sensors acquire the light reflected or emitted from the planet’s surface in 13 spectral bands and at 10, 20- or 60- meters spatial resolutions (37). After 6 years of regular complete acquisitions, these datasets offer new opportunities to understand the landscape in which host and vector proliferate and interact. The micro-scale level at which it is now possible to study the presence of mosquitoes is relevant for both larval and adult stadium, considering the relatively short flight distance (usually a few hundred metres) during its adult lifespan (38).

This substantial volume of data calls for additional methods of analysis and deep learning, exploiting patterns and dependencies in the provided raw data to extract information, without relying on specific *a priori* hypothesis (32). Deep neural network (DNN) architecture is composed of neurons, synapses, weights, biases, and functions, coarsely mimicking the functioning of the human brain: DNN uses multiple layers (intermediate understanding) to progressively succeed in its task, i.e., extract higher-level features from the raw input (39). Convolutional neural networks (CNNs) are a type of deep neural network designed primarily for pattern recognition in visual data. They are widely used in computer vision applications, such as image recognition and object detection (40): in image processing, lower layers may identify edges, while higher layers may identify more complex and abstract features (e.g., digits or letters or faces). The hidden layer learns features from the input images (by means of a set of parameters), and it is subject to a function (non-linear activation) which reduces computational complexity while retaining multi-scale information. The sequence of these layers and functions allows the modeling to learn increasingly complex and abstract features at different scales of the original image (39). During the DNN training phase, the parameters (weights, biases) are iteratively adjusted so that the output layers of the DNN best approximate the ground-truth target (41).

The application of deep learning to high spatial resolution data to predict the potential distribution of mosquito species (42) or to predict outbreaks of VBDs (43), is in its nascent stages (44). Few studies deal with animal, disease and plant distribution modeling through DNN (45–47).

In this paper, we present the combination of deep learning methods and high spatial resolution (20 m) remotely sensed imagery with naive spectral bands applied to the distribution of presence/absence *Cx. pipiens* species occurrences in central Italy (whose territory covers a wide range of environmental conditions, i.e., wide domain of eco-climatic values). Aims of this paper were:

- To provide pictures (maps) of the spatio-temporal distribution of *Cx. pipiens* abundance in central Italy (Abruzzo and Molise regions), as derived from a 4 years in-field sampling.
- To identify suitable areas in space and time for *Cx. pipiens* distribution in Abruzzo and Molise regions.
- To propose a new robust deep learning model able to “predict” in advance the presence of the species.

## 2 Materials and methods

### 2.1 Entomological data

#### 2.1.1 In-field collection

**Figure 1:**
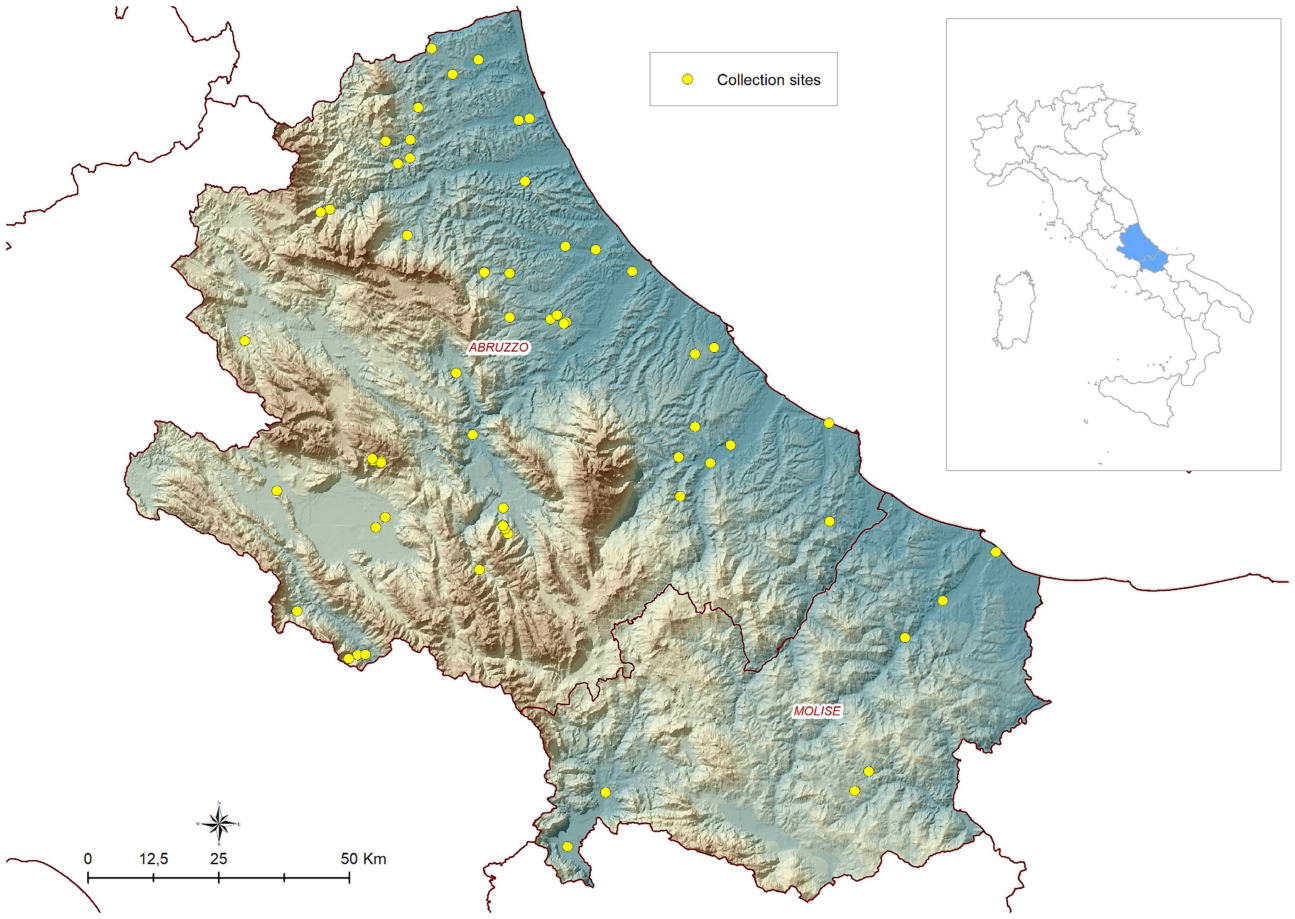
Geographical location of the study area (Abruzzo and Molise regions in central Italy) and distribution of entomological sites.

Four seasonal campaigns of field collections were carried out in the years 2019, 2020, 2021 and 2022 in the Abruzzo and Molise regions in central Italy. In 2019, activities were part of a research project funded by the Ministry of Health and aimed to map the local mosquito avifauna (IZSAM 01/18 RC). The activities carried out in 2020, 2021 and 2022 were part of the 2020–2025 Integrated Surveillance and Response Plan for Arboviruses (PNA) (https://westnile.izs.it/j6_wnd/ministeriale, accessed on November 10, 2022). The presence/absence and abundance of *Cx. pipiens* were collected on a weekly/biweekly basis during the vector season (between April and November) at 56 sites in 2019, and at 17/18 sites in the subsequent years (Figure 1 shows the study area and the location of the sites).

The locations of the mosquitoes’ traps were distributed across the territory based on a two-step process. Initially, at a macro-scale, areas for trap placement were chosen to include different ecoregions representing a broad variety of climatic and environmental conditions in the two regions (25).

Subsequently, at a local scale, mosquito collection sites were chosen based on the presence of mosquito breeding sites. Before starting entomological activities, all sites were investigated to identify the optimal locations for trap placement. Mosquito collections were performed using CDC type traps baited with both light and dry ice, placed at about 1.5 m above the ground and activated just before sunset. The following morning, the collected insects were labelled and transferred to the laboratory, where mosquitoes were counted and morphologically identified using identification keys (48). A detailed description of entomological in-field activities is reported in De Ascentis et al. (14).

#### 2.1.2 Ground-truth data collation

Entomological data were structured in a geodatabase where the abundance was dichotomized to focus on the spatio-temporal distribution of the presence/absence of *Cx. pipiens* species. We labelled the data into positive and negative collections:

- “Positive collection” is defined as the night of catch at a trap location where at least one *Cx. pipiens*’ specimen was recorded.
- “Negative collection” refers to the night of catch when no *Cx. pipiens* was captured. Since the field activities were performed during the vector season and not evenly distributed throughout the year, the ground-truth database was theoretically unbalanced favouring positive collections. For this reason, pseudo-absence data were generated on a biweekly basis in the same site locations, assuming the absence of *Cx. pipiens* in December and January (15, 49). Although *Cx. pipiens* can overwinter at our latitudes (5), the population numbers are very low during cold months, allowing us to consider collections made in those months as negatives. Summarising, “negative collection” refers to no *Cx. pipiens* captured during the vector season, or to pseudo-absence collections generated in winter months.

### 2.2 Earth Observation data

The EO datasets considered in the study were:

- Copernicus Sentinel-2 spectral bands at 20 m spatial resolution
- MODIS Land Surface Temperature and Emissivity (LST).

The Sentinel-2 mission is characterized by acquiring data with 13 bands in the visible, near infrared, and short-wave infrared part of the spectrum. Data is acquired with a swath width of 290 km, and every 5 days (revisit time). In terms of spatial resolution, bands B02, B03, B04, B08 are acquired at 10 metres; bands B05, B06, B07, B8A, B11 and B12 at 20 metres; bands B01, B09, B10 at 60 metres (50).

For this research, the 20-metres spatial resolution was chosen as the reference resolution, as it offers a balance between capturing fine-scale relationships between environmental conditions and *Cx. pipiens*—and computer processing times.

Copernicus distribution service provides level-2A output image products resampled and generated at an equal spatial resolution for all bands (10 m, 20 m or 60 m), regardless of the acquisition resolution (https://sentinels.copernicus.eu/web/sentinel/user-guides/sentinel-2-msi/processing-levels/level-2 last access on March 19, 2024). The 20-metres spatial resolution images provided by Copernicus service are: AOT, B02, B03, B04, B05, B06, B07, B11, B12, B8A, CLD, SCL, SNW, TCI, WVP. We considered these bands, excluding TCI and SCL: TCI was excluded as it is a combination of bands B04, B03, B02; and SCL - scene classification was excluded as it is a derived product from the other bands developed by ESA to distinguish between cloudy pixels, clear pixels and water pixels. Table 1 reports the mentioned bands and their main properties.

**Table 1:** List of Sentinel-2 spectral bands, brief description and main spectral properties.

Band	Description	Central wavelength (nm)	Bandwidth (nm)	Acquisition spatial resolution (m)
**Spectral bands**				
**B01**	Aerosol. For aerosol detection	442.7 (S2A) 442.3 (S2B)	20	60
**B02**	Blue channel. Sensitive to vegetation senescing, carotenoid, browning, and soil background; atmospheric correction (aerosol scattering)	492.4 (S2A) 492.1 (S2B)	66	10
**B03**	Green channel. green peak; sensitive to total chlorophyll in vegetation	559.8 (S2A) 559.0 (S2B)	36	10
**B04**	Red channel. maximum chlorophyll absorption	664.6 (S2A) 664.9 (S2B)	31	10
**B05**	Position of red edge; consolidation of atmospheric corrections—fluorescence baseline	704.1 (S2A) 703.8 (S2B)	15 (S2A) 16 (S2B)	20
**B06**	Position of red edge, atmospheric correction; retrieval of aerosol load	740.5 (S2A) 739.1 (S2B)	15	20
**B07**	Vegetation of red edge, LAI, edge of the NIR plateau	782.8 (S2A) 779.7 (S2B)	20	20
B08	NIR: plateau; shorelines and biomass content	842	115	10
**B8A**	NIR plateau; sensitive to total chlorophyll, biomass, LAI, and protein; water vapor absorption reference; retrieval of aerosol load and type	864.7 (S2A) 864.0 (S2B)	21 (S2A) 22 (S2B)	20
B10	SWIR and cirrus: cirrus cloud detection	1,375	30	60
**B11**	SWIR Sensitive to lignin, starch, and forest aboveground biomass; snow–ice–cloud separation	1613.7 (S2A) 1610.4 (S2B)	91 (S2A) 94 (S2B)	20
**B12**	Assessment of Mediterranean vegetation conditions; distinction of clay soils for the monitoring of soil erosion; distinction between live biomass, dead biomass, and soil (e.g., for burn scars mapping)	2202.4 (S2A) 2185.7 (S2B)	175 (S2A) 185 (S2B)	20
**Additional datasets**				
TCI	True Colour Image (based on bands 4,3,2)			
**AOT**	Aerosol Optical Thickness map (at 550 nm)	(51)		
**WVP**	Scene-average Water Vapour map	(52)		
SCL	**Scene classification layer—classification of Sentinel-2 data as results of ESA’s Scene classification algorithm	(53)		
**Quality index**				
**CLD**	Raster mask values range from 0 (for high confidence clear sky) to 100 (for high confidence cloudy)			
**SNW**	Raster mask values range from 0 for high confidence NO snow/ice to 100 for high confidence snow/ice			

The bands used in this study are highlighted with bold.

The Level-2A (Bottom-Of-Atmosphere reflectances in cartographic geometry) Sentinel-2 images covering the area of interest for the years 2019, 2020, 2021 and 2022 were downloaded using a script based on the Sentinelsat (https://sentinelsat.readthedocs.io/en/stable/ accessed on July 05, 2023), open source Python package connecting to the Copernicus Open Access Hub platform API, via https://scihub.copernicus.eu/ (accessed on July 05, 2023). The Sentinel-2 tiles covering the study area were T33TUG, T33TUH, T33TVG, T33TVH, T33TVF in the orbits R122 and R079; in case of overlapping images for the same date, only one dataset was retained.

For each downloaded data package, only the images of bands with a spatial resolution of 20 metres were selected and extracted by the script. Band values range in [0,1] and they were not subjected to any band aggregation or elaboration. Furthermore, no filtering was applied for cloud coverage, shadows or other pixel value selection, aiming to let the deep learning algorithms to independently discern the utility of pixels, thus avoiding time-consuming pre-processing operations.

Regarding temperature, the product considered was the Land Surface Temperature and Emissivity (LST) of the Earth (MOD11A2 version 061), derived from the Moderate Resolution Imaging Spectroradiometer (MODIS) sensor, by the Terra platform of NASA (54). The layer used is daytime temperatures (LST_Day_1km), at a spatial resolution of 1 km, and a temporal resolution of 8 days. Each pixel’s value is an average of all corresponding LST pixels collected during the 8-day period. LST data were downloaded from the LP DAAC User Services repository, accessible from https://e4ftl01.cr.usgs.gov/MOLT/ (accessed on July 05, 2023) through R, extracting the LST_Day_1km layer in GeoTIFF files and transforming the pixel values from Kelvin to Centigrade degrees. In case of empty pixels in the LST rasters, due to cloud cover or invalid values pre-filtered at LP DAAC, a gap-fill procedure was applied. This procedure adaptively considers surrounding pixels (in space and time), ranks the images, estimates the empirical quantiles, for characterising missing values and predicts the value through quantile regression (55).

LST rasters were nearest neighbor resampled to match the chosen spatial resolution of 20 metres (as Sentinel-2 bands).

#### 2.2.1 EO datacube collation

Sentinel-2 and LST images with a 20-meter spatial resolution were cropped around the site locations using a bounding box with sides of 4,480 metres, through a GDAL (GeoData Abstraction Library, https://gdal.org/) based Python script. The buffer size accounted for the flight range variability of *Cx. pipiens* (15, 38, 56, 57), as well as for encompassing the landscape in the surroundings of the traps, which influence the vector life cycle (58).

The resulting 224 × 224 pixel PNG (portable network graphics) images, common input size in CNN architectures (39), were generated for each Sentinel-2 revisit time from 2019 to 2022 and across all spectral bands, along with LST raster images.

Three EO datacubes were created, corresponding to the three following scenarios and modeling.

1. Baseline model. In each site, we consider the images at the time of mosquito collection. We consider the Sentinel-2 acquisition preceding the collection date and spatially overlapping the site (bounding box). No temperature or information from other sites are included.
2. Multitemporal model. This model considers the sequence of local conditions occurring approximately in the 2 months preceding the mosquito collection. This scenario considers the variability in environmental conditions during the *Cx. pipiens* main life cycle and potential changes in habitats (such as the creation or disappearance of larval habitats). This timeframe also accommodates any temporal disparities between satellite image acquisitions (occurring every 5 days) and weekly/biweekly mosquito catches. For each date and site, each collection is associated with a series of 10 Sentinel-2 acquisitions preceding the mosquito collection. No temperature, no information from other sites are considered.
3. MultiAdjacency Graph Attention Network (MAGAT) model. This model considers the Sentinel-2 image and the relationship with nearby geographical sites. In this scenario, we assume that areas with similar climatic and environmental conditions are potentially able to sustain similar mosquito patterns. To exploit this information, we consider the acquisitions made around the sites and arrange them according to a graph structure: the nodes represent the sites and the edges represent similarities between those sites. Similarities are in terms of temperatures (MODIS daytime LST) and geographical distance (Haversine).

**Figure 2:**
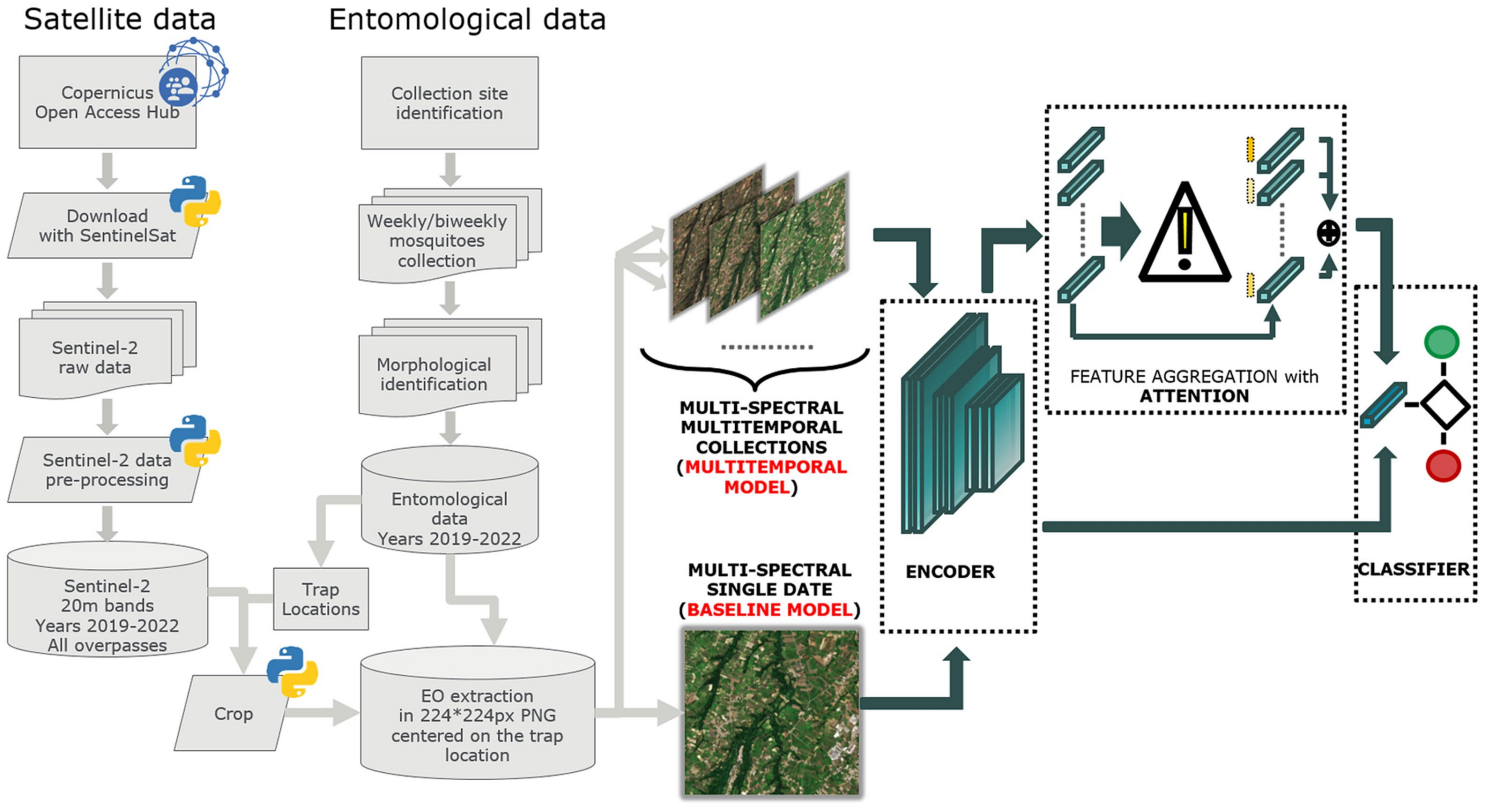
Flow chart of the process adopted to produce Sentinel-2 and entomological data and to develop the baseline and multitemporal models.

**Figure 3:**
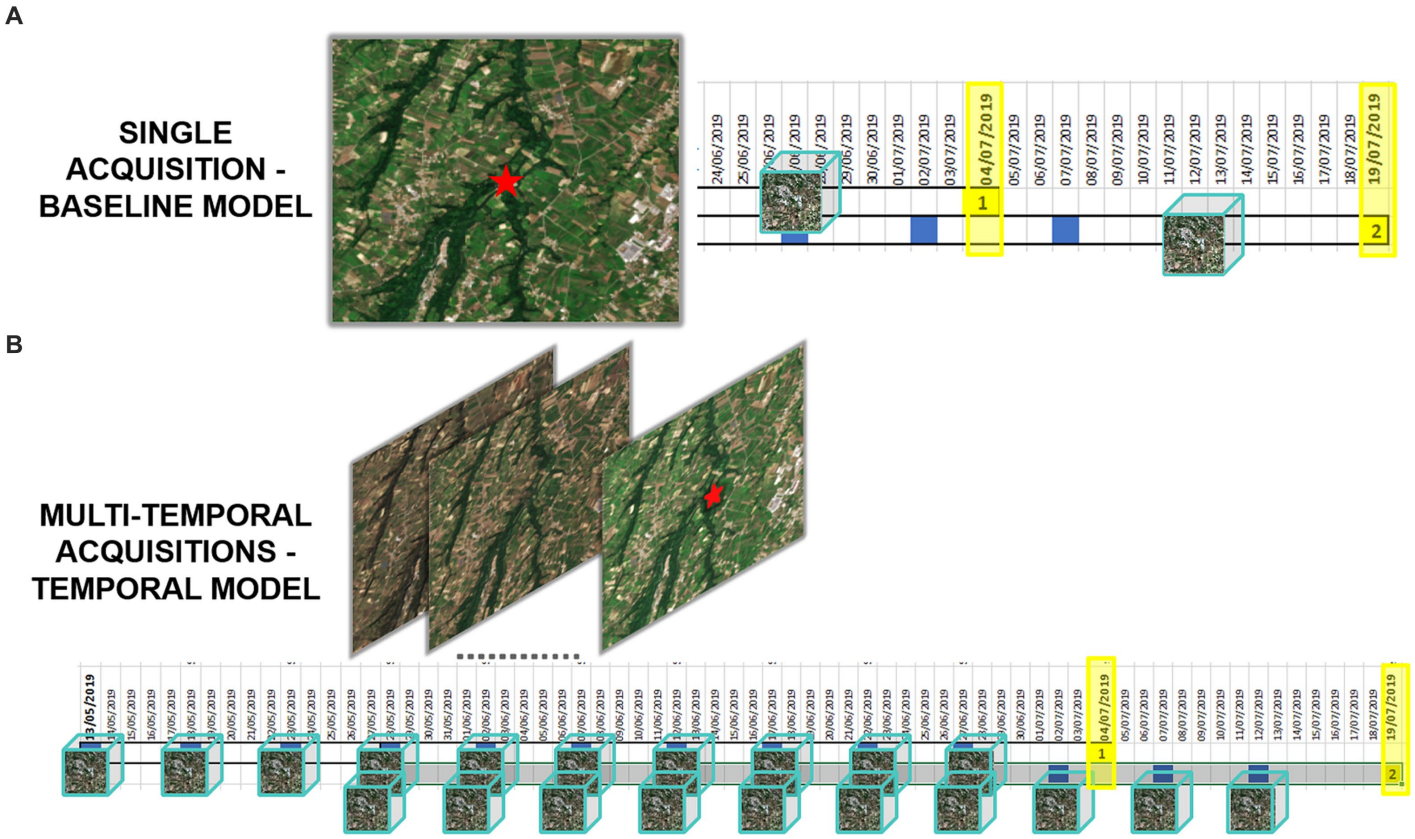
Graphical representation of Sentinel-2 data associated with mosquito collections, in the baseline **(A)** and multitemporal models **(B)**. Yellow highlighted dates are the entomological collection dates, the blue squares represent the Sentinel-2 overpass over the area, the EO datacubes are represented as cubes.

**Figure 4:**
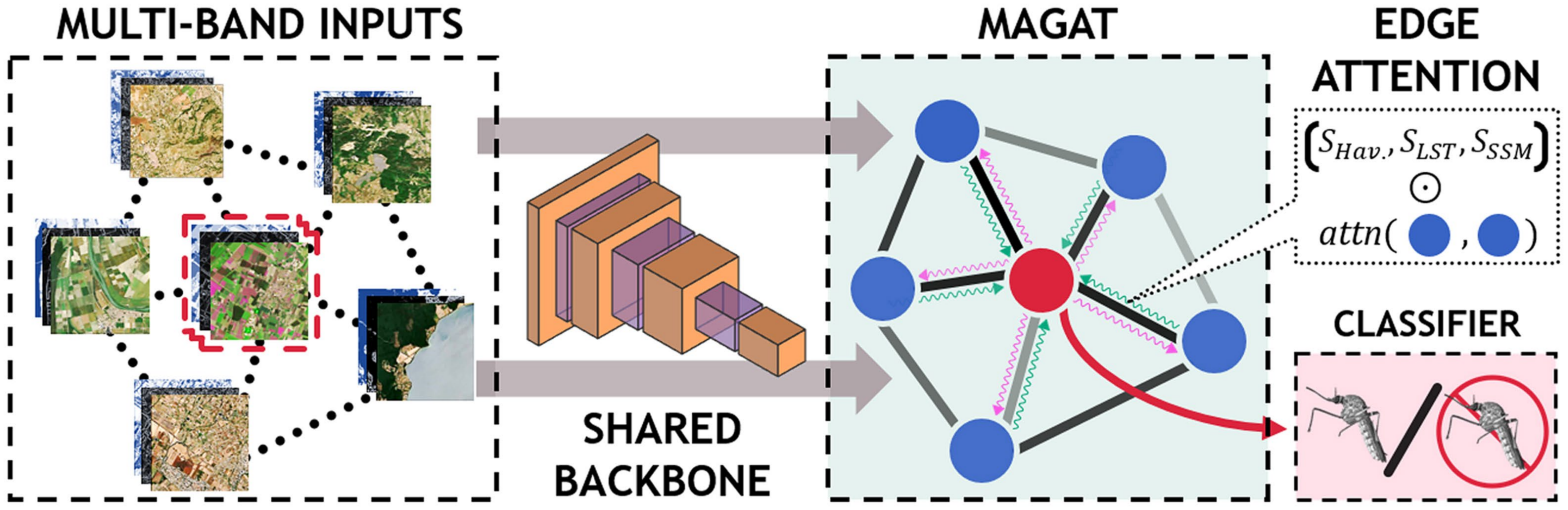
Graphical representation of the MAGAT model.

Figure 2 illustrates the process for acquiring and processing Sentinel-2 data, integrating entomological data, and developing baseline and multitemporal models. In detail, the association between Sentinel-2 data and mosquito collections is presented in Figure 3 for both baseline (A) and multitemporal (B) models. Figure 4 shows the schema for the MAGAT model.

### 2.3 Modeling

#### 2.3.1 Overall approach

The research question aimed to predict areas in central Italy suitable for the presence/absence of *Cx. pipiens*, exploiting Sentinel-2 and other remotely sensed data combined with deep learning algorithms. The research question was formulated as a binary classification task, consisting in the prediction of the vector’s presence/absence. To this end, the entomological data on *Cx. pipiens* at the sites and dates of collection, along with the pseudo-absence data, were paired with a series of *n* Sentinel-2 images closest-in-time before the collection date. Entomological data was splitted into separate and non-overlapping sets: a training subset (80% of the observations) used for model training, and a test subset (20%) for model validation (further details in section 2.3.4).

The common denominator of our models is the use of a convolutional neural network (CNN). These networks employ the convolution operation in at least one layer, where learnable filters (or kernels) slide across the input image, performing element-wise multiplication to extract features. Through a series of convolutional layers, CNNs can process an input image and extract a smaller high-level representation, effective at isolating important elements of the input. The convolution operation allows the detection and analysis of spatial hierarchies within images. The ResNet (Residual Network) family (59) of CNN architectures was used in this research: specifically, we exploited the ResNet-18 backbone, consisting of 18 convolutional layers, extensively employed in the field (39).

#### 2.3.2 Baseline and multitemporal deep learning architectures

The baseline model employs features extracted from a single multi-band image (Sentinel-2 acquisition) by the convolutional backbone, corresponding to the closest acquisition before mosquito collection (Figure 3A). It then performs a linear transformation to produce the final output probabilities. The algorithm delivers the probability of each collection being positive or negative.

The multitemporal model considers the most recent sequence of multi-band image acquisitions (Figure 3B). Ten Sentinel-2 acquisitions, one every 5 days, were associated with each date of mosquito collection, covering approximately 2 months before the date of field collection. Each acquisition is processed independently by the backbone and aggregated by means of an “attention” module. The latter produces a single average representation, which is weighted by the estimated importance of each multi-band image. Specifically, we denote with h the features produced by the CNN for each image; the importance $a\left(h\right)$ is then computed as:


$$a\left(h\right)=W_{w}^{T}\left[\sigma \left(W_{v}^{T}h\right)⊙\tanh\left(W_{u}^{T}h\right)\right]$$

where $W_{w},W_{v},W_{u}$ are learnable matrices, *σ* is the sigmoid operation, ⊙ denotes the element-wise product (also known as the Hadamard product), and $W^{T}h$ denotes the matrix-product between W and h . Once aggregated, the final result is obtained by aa linear transformation.

##### 2.3.2.1 Prediction in the future

For both models, we tested a series of temporal lags between the date of acquisition of the multi-band EO datacube and the date of the mosquito’s collection: instead of using the first available Sentinel-2 acquisition preceding the collection date, we use the first one available *t* days before the collection, with t = days of the temporal lag and *t* = 0, 5, 10, 15, 25, 50. This temporal lag simulates the scenario where EO data is collected and processed ahead of *Cx. pipiens* presence, to alert health authorities in advance about the risk of vector presence.

#### 2.3.3 MAGAT architecture

In our third scenario, we aim to investigate if areas with similar environmental conditions and geographically close could exhibit similar mosquito outcome (presence/absence).

To this end, we model the relationships in the data with a graph structure: the nodes represent the geographical sites and the edges denote similarities between them. We include data on temperature differences (MODIS daytime LSTD), and geographical distances (Haversine) between the sites.

The multi-band satellite images are independently processed by the CNN to obtain higher level representations for the inputs (schematic representation is shown in Figure 4).

The outputs of the CNN are then arranged as a graph and processed using a graph neural network (GNN). The GNN computes a single representation by sharing the information between the nodes based on the information on the edges (Figure 4). In particular, as for the multi-temporal model, we denote with $h_{i}$ the features extracted by the *i*-th multi-band image (node). From these, our GNN first computes the similarity between each pair of nodes as:


$$s\left(h_{i},h_{j}\right)=\exp\left\{\mathrm{lReLU}\left(p^{T}\left[Vh_{i}∥Vh_{j}\right]\right)\right\}$$

where p and V are learnable matrices, || indicates the concatenation operation, and lReLU is the leaky ReLU (Rectified Linear Unit) activation function. We then use such similarities to extend the edge information. The computation then follows the classical aggregation of the graph convolutional network (GCN) (60) to compute a single aggregated representation for all sites. To account for the multiple relationships that exist between nearby locations (in our case, temperature and geographic distance), we repeat the procedure described above for each environmental feature. The output of this operation, is then processed by a linear transformation to obtain the final classification result.

#### 2.3.4 Model evaluation

The group of entomological sites was divided in two subsets: a training subset (80% of the observations) used for model training, and a test subset (20%) used by the model to evaluate its performances.

The dataset was divided using the stratified k-fold cross validation technique (with *k* = 5), ensuring a balanced representation of sites across each fold while maintaining uniformity in the categorical outcomes within each subset. This stratified approach was crucial for maintaining the validity of the model evaluations, allowing us to avoid misleading results by segregating sites (and their associated imagery for each collection) to ensure no repetitions between the two subsets. For each site and collection, its related imagery was included in the model following the previously defined criteria (baseline, multitemporal, MAGAT models and time lags).

Following the approach of Vincenzi et al. (61), the models were pre-trained on a separate—and larger—set of data, followed by fine-tuning on our dataset. This strategy is called knowledge transfer and its aim is to achieve good results even in presence of a reduced number of labelled examples. Specifically, we exploit a pre-training strategy targeting the RGB bands (B04, B03, B02 of Sentinel-2), utilizing the ImageNet dataset (62) as a starting point.

The metrics used for evaluation were: sensitivity, specificity and F1 score. Sensitivity measures the model’s ability to detect true positives (in our study, it correctly identifies mosquito presence collections); specificity assesses its accuracy in identifying true negatives. The F1 score metric combines sensitivity and specificity.

#### 2.3.5 Software used for modeling

The ESRI^©^ ArcMap version 10.8.1 software (Redlands, ESRI. ArcGIS Desktop: Release 10. 2011) was used for geographical manipulation of vector and raster data, and for map creation. Entomological data were processed through Microsoft Access and Excel. The processing software of EO data was described in the dedicated sections and the code for download and preprocessing is available on GitHub public repository at https://github.com/IZSAM-StatGIS/spotting_cp_satellite.

To build and train the DNN, we relied on the Numpy (63) and PyTorch (64) libraries, with performance metrics computed using tools from Scikit-Learn.

The code implementing the deep learning architectures is publicly available on GitHub public repository at https://github.com/loribonna/release_frontiers_wnv.

## 3 Results

### 3.1 Entomological data

Field collections were conducted across four seasons in the years 2019, 2020, 2021, and 2022. During these campaigns, the presence/absence and abundance of *Cx. pipiens* were systematically recorded on a weekly/biweekly basis at 60 sites during the vector season. Over the 4 years of entomological surveys, a total of 2,158 field collections were performed in the sites (1,336 with at least one specimen caught, 822 negative). Additionally, 397 pseudo-absence collections for winter months were incorporated into this dataset to augment the total number of collections. These collections took place in the Abruzzo and Molise regions of central Italy: Figure 1 shows the study area.

**Figure 5:**
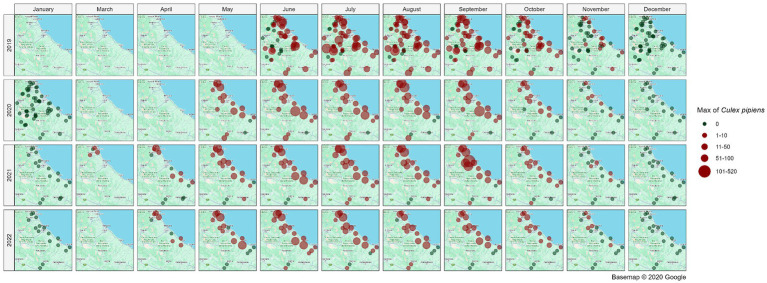
Spatial distribution of collection sites across the spatio-temporal area of interest; the abundance of *Cx. pipiens* (maximum number of mosquitoes catched per site, month and year in a single night of collection) is shown. Source of background map: Google maps.

Figure 5 illustrates the spatial distribution of mosquito collection sites within the area of interest, providing a monthly and yearly breakdown of the maximum number of *Cx. pipiens* specimens captured in a single collection. Overall, the map illustrates a distinct temporal pattern in the abundance of *Cx. pipiens* throughout the year. The abundance of *Cx. pipiens* remained consistent across the 4 years at different sites: sites with high abundance in a given year confirm that level in the following years, while sites characterized by low abundance tended to maintain their lower levels over time.

**Figure 6:**
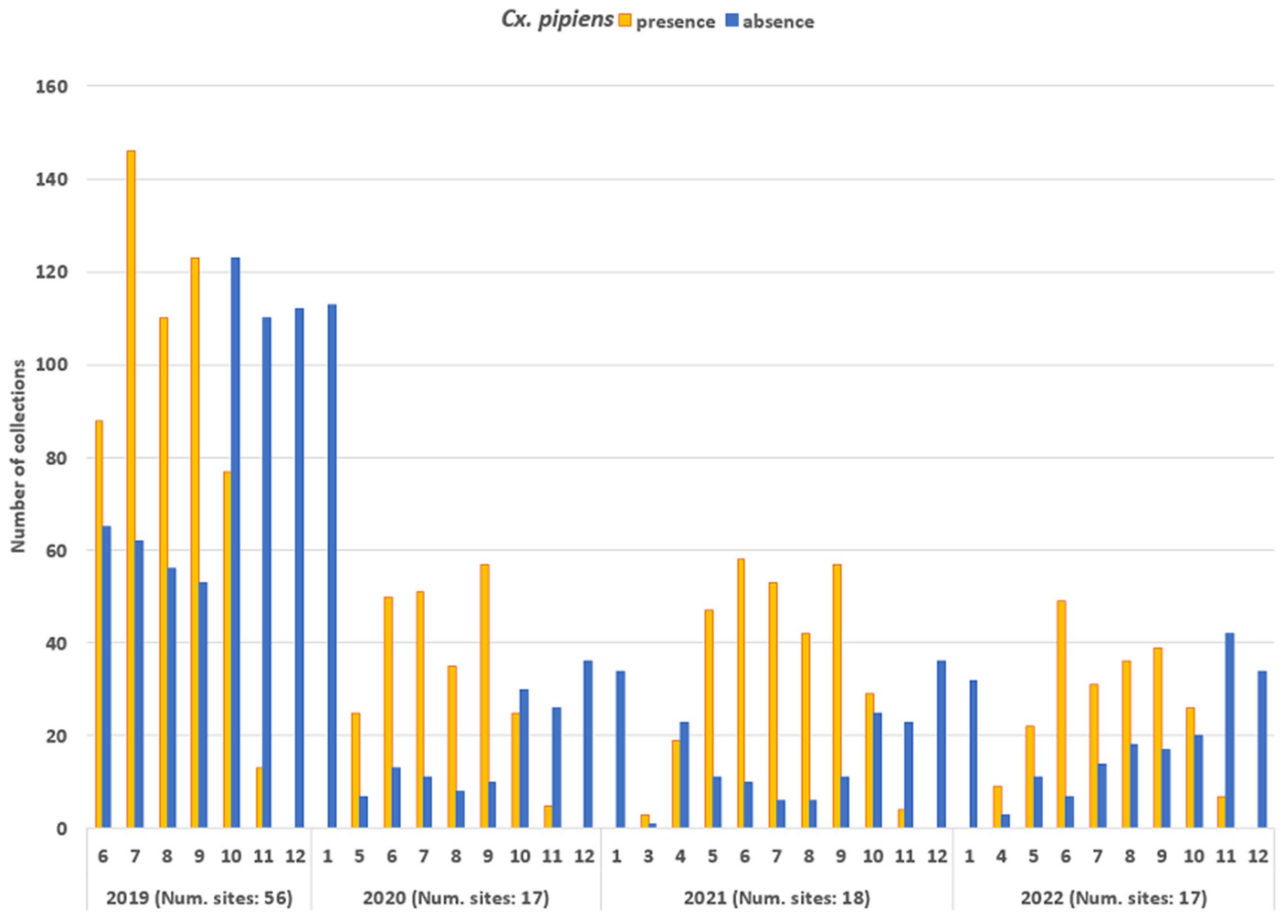
Temporal distribution of the number of entomological collections, distinguished in collections with *Cx. pipiens* presence (orange bars) and *Cx. pipiens* absence (blue bars).

The entomological dataset was subsequently dichotomized, categorising each collection as either vector presence (when the number of specimens exceeded 1) or absence (when the number of specimens was zero): the total ground-truth database comprised 2,555 records. Figure 6 shows the temporal distribution of the sampling records (collections). In 2019, the number of sampling sites was higher than in subsequent years (56 compared to 17-18-17, respectively) resulting in a higher total number of collections. The mean number of collections per site was as follows: for 2019—18.3, for 2020—20.8; for 2021—23.8; for 2022—20.6. Collections were mainly conducted during the vector season, between April and November: in 2019, collection started in June (due to logistical set up of all field activities) and in the 2020, activities started in May.

### 3.2 Earth Observation data

**Figure 7:**
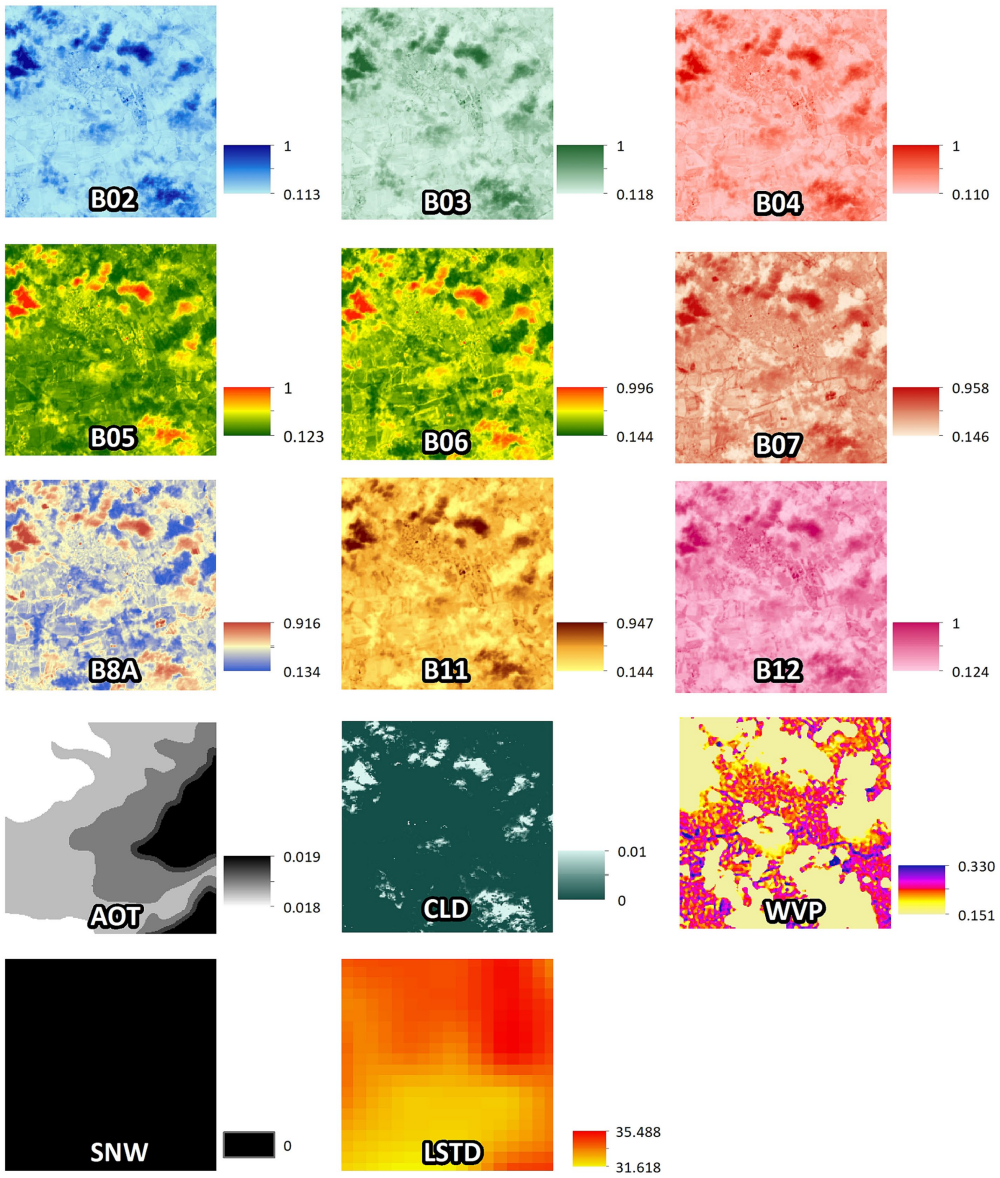
Spectral bands of a Copernicus Sentinel-2 image for the patch of 224 × 224 pixels around the collection site TE05_NER, acquisition date 2022-05-30, tile T33TUH. Last picture refers to daytime (LSTD) land surface temperatures of the period 2022-05-25 to 2022-06-01. Source of background map: Google maps.

The Copernicus Sentinel-2 spectral bands, including B02, B03, B04, B05, B06, B07, B8A, B11, B12, along with additional data such as AOT (Aerosol Optical Thickness), CLD (Cloud Coverage), WVP-B09 (Water Vapor) and SNW (Snow Cover) were inputted in the deep models (Table 1). Furthermore, daytime temperature derived MODIS LSTD were included in the MAGAT model. Figure 7 provides an example of the imagery used as input of the models, focusing on one of the collection sites (TE05_NER) acquired on May 30, 2022 (LSTD corresponding to the period from May 25, 2022, to June 1, 2022). Table 2 reports the number of remotely sensed images processed from Copernicus Sentinel-2 and MODIS LST_Day_1km.

**Table 2:** Number of collection sites per year and EO data volume.

		Year			
		2019	2020	2021	2022
Number of sites		56	17	18	17
*Earth Observation datasets*					
Copernicus Sentinel-2	Dimension of the dataset, i.e., number of spectral bands (B02, B03, B04, B05, B06, B07, B8A, B11, B12, AOT, WVP, CLD, SNW)	13	13	13	13
Level 2	Median number of satellite overpasses on the same site (average number of datacubes per year per site)	72	73	73	73
	Total number of Sentinel-2 imagery (number of bands * number of satellites overpasses * number of sites)	52,728	16,133	16,939	17,296
MODIS LSTD	Median number of images per site	46	46	46	46
	Total number of MODIS imagery (number of 8-days product * number of sites)	2,576	782	828	782
	Yearly number of EO patches	55,304	16,915	17,767	18,078
	Overall number of EO patches	108,064			

### 3.3 Model performances

The model evaluation after training phase was done using 5-fold cross validation, wherein the data was split into 5 non-overlapping folds.

**Figure 8:**
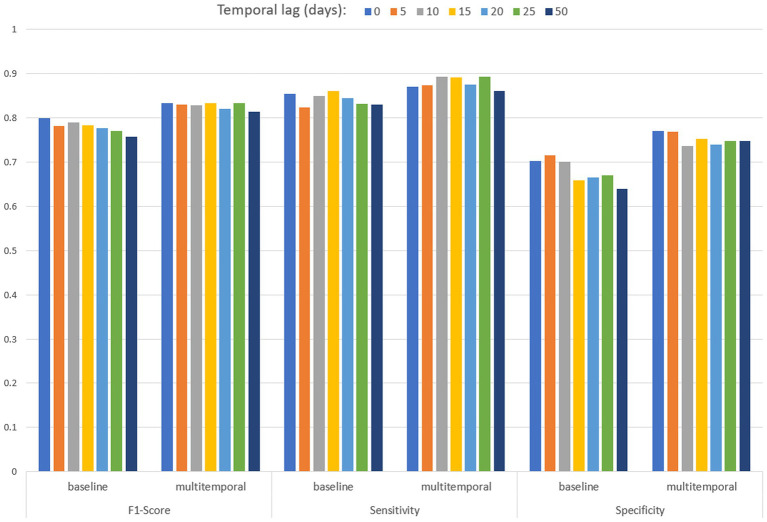
Performances of the baseline and multitemporal models by temporal lag.

Table 3 and Figure 8 present the performances of both baseline and multitemporal models. These performances are measured using the metrics F1 score, sensitivity and specificity, across different temporal lags, that is the days between the EO imagery acquisition and the date of mosquito collection. All F1 score values are higher than 0.758 in the baseline model, and are higher than 0.814 for the multitemporal model. Sensitivity values are higher than 0.824 for any temporal lag of the baseline model; sensitivity values are higher than 0.861 for any temporal lag of the multitemporal model. In the baseline model, the highest specificity performance is 0.716, achieved for a temporal lag of 5 days; specificity values are higher than 0.736 for any temporal lag of the multitemporal model. Results suggest that considering multiple images enhances model robustness, particularly in specificity, with a significant effect also on the F1 score.

**Table 3:** Performances of the baseline (including one Sentinel-2 multiband image) and multitemporal (including 10 Sentinel-2 multi-band images) models by temporal lag (days back in time since mosquito collection).

Temporal lag (days)	Baseline			Multitemporal		
	F1 score	Sensitivity	Specificity	F1 score	Sensitivity	Specificity
0	0.799	0.855	0.703	0.833	0.871	0.770
5	0.782	0.824	0.716	0.830	0.873	0.769
10	0.790	0.849	0.701	0.828	0.893	0.736
15	0.783	0.860	0.659	0.834	0.892	0.752
20	0.777	0.845	0.666	0.820	0.875	0.739
25	0.770	0.831	0.670	0.833	0.893	0.747
50	0.758	0.830	0.640	0.814	0.861	0.748

Table 4 reports the performances of the MAGAT model. The highest performances are reached for the closest temporal lag to the mosquito collection, and they are closest to baseline metrics, lower than multitemporal ones.

**Table 4:** Performance of the MAGAT model (including one Sentinel-2 multiband image, geographical distance, LSTD) by temporal lag (days back in time since mosquito collection).

Temporal lag (days)	F1 score	Sensitivity	Specificity
0	0.809	0.844	0.748
5	0.802	0.837	0.721
10	0.796	0.826	0.735
15	0.787	0.824	0.708
25	0.747	0.765	0.709
50	0.635	0.644	0.630

**Figure 9:**
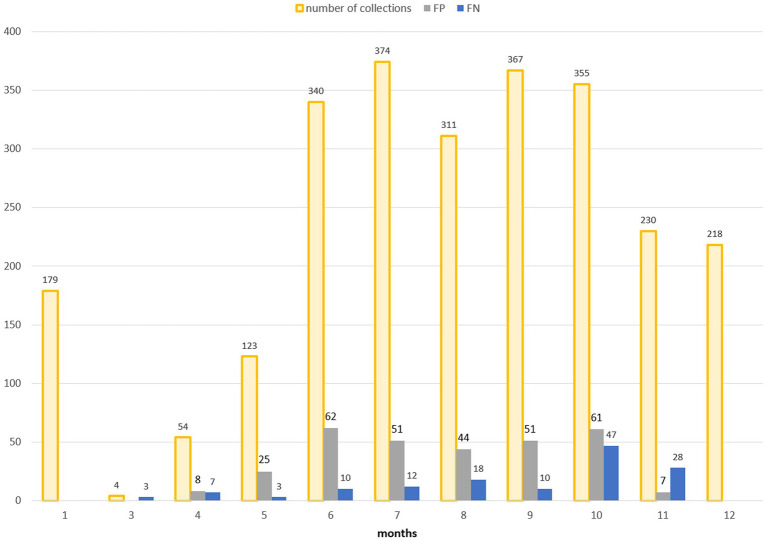
Distribution in time (months on *x*-axis) of false positives (FP, grey bars) and false negatives (FN, blue bars) in the multitemporal model with lag3 (15 days). The total number of collections is additionally reported (orange bars).

For a more in-depth analysis of the model’s behavior, the multitemporal model is considered as it has the highest performances. Figure 9 shows the classification results obtained using the multitemporal model with a temporal lag of 15 days: total number of collections and misclassified collections are reported on a monthly basis.

**Figure 10:**
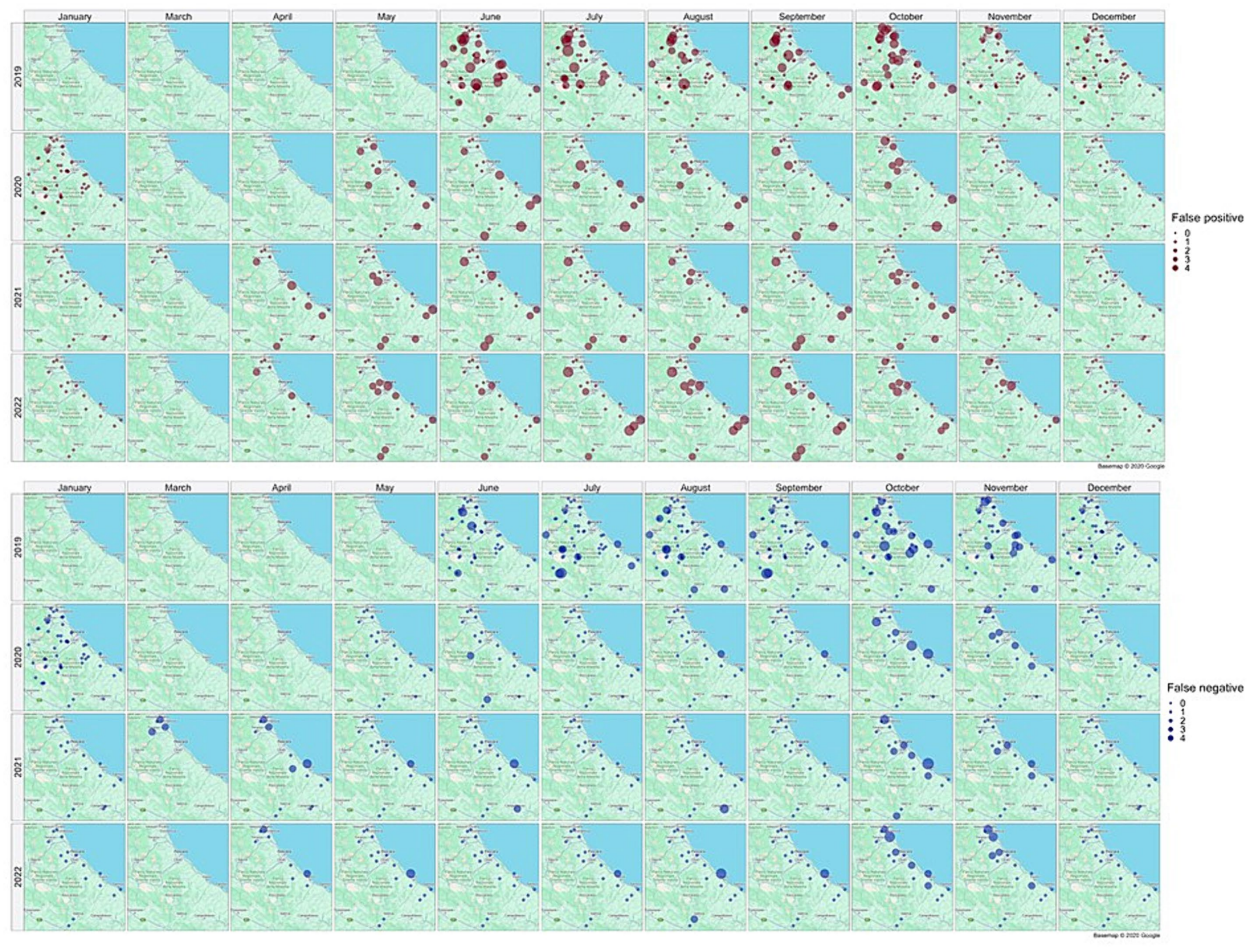
Distribution in space and time of false positive (brown dots) and false negative (blue dots) collections. Source of background map: Google maps.

Figure 10 illustrates the spatial distribution of false positive (brown dots) and false negative (blue dots) collections, providing a monthly and yearly breakdown.

**Figure 11:**
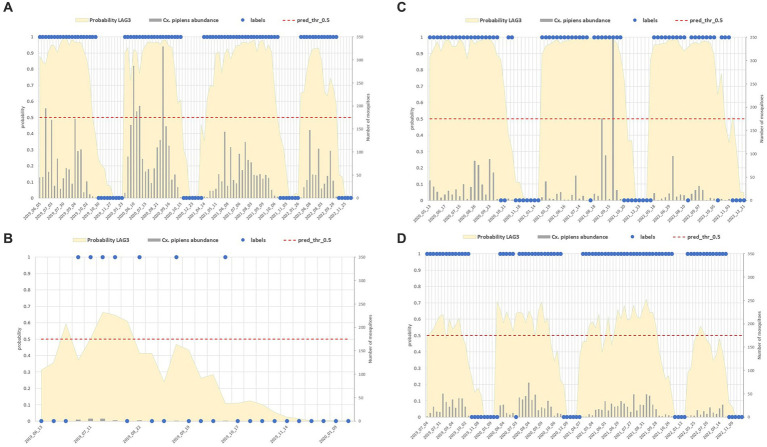
Comparison between field data (abundance of the mosquito population reported in grey bars, right axis, and its dichotomous classification in blue dots) and model predictions (yellow surface) in four sites with different characteristics: mosquito abundant site **(A)**, low abundance **(B)**, sites with misclassified collections **(C,D)**.

Figure 11 shows different site examples in which the *Cx. pipiens* presence and absence is correctly estimated (Figures 11A,B) and sites in which misclassification occurred (Figures 11C,D).

False positive estimates are prevalent during summer months (Figures 10, 11C,D) and are often between true positive collections. In these occurrences, the model keeps forecasting positive environmental conditions for mosquitoes’ presence while unpredictable factors, such as weather variations during night collection (wind or rain), malfunctioning of trap equipment, or insecticides treatment near the collection sites, may have influenced these field collections. Additionally, some false positives are observed in “transitional months,” specifically June and October, when mosquito population abundance is either increasing or decreasing. During these months, natural fluctuations in the number of specimens caught are common.

Spring and, particularly, autumn months emerge as crucial periods for false negatives (FN), signifying instances where the model fails to detect the presence of *Cx. pipiens*. These instances are depicted by the blue bars in Figure 9 and blue dots in Figure 7. When focusing exclusively on false negatives (138 collections out of 2,555), the 44.20% of them correspond to instances where a collection involved only one specimen of *Cx. pipiens*; the 80.43% of FNs refers to collections with no more than 5 mosquitoes caught. Table 5 provides the entire distribution of FN values and their respective frequencies.

**Table 5:** Distribution of false negative values by abundance of species: the majority of FN predictions occur when the number of mosquitoes is low (1 to 5 specimens per collection).

Abundance of *Cx. pipiens* in a single collection (number of specimens)	Frequency of collections with the mentioned mosquito abundance	% cumulative
1	61	44.20%
2–5	50	80.43%
6–10	10	87.68%
11–20	7	92.75%
21–30	5	96.38%
31–40	3	98.55%
41–50	2	100.00%

## 4 Discussion

This study demonstrates successful synergy among entomological field activities, new high-resolution satellite imagery (Sentinel-2) and advanced algorithms (AI Deep Learning) in predicting the presence/absence of *Cx. pipiens* in central Italy. The algorithms were trained using a dataset that combines 4 years of mosquito field collection data (2019–2022) with environmental information from multispectral bands at 20 m spatial resolution in the surroundings (4480 × 4480m) of each trap location.

Over 4 years of entomological activity, in Abruzzo and Molise regions, *Cx. pipiens* was found in every province of the study area, with different environmental conditions across its territory (25). This result is not surprising as *Cx. pipiens* is able to adapt to a wide variety of habitats and this species has already been reported as one of (if not the) most abundant mosquito species in Italy (5). Consistently with other studies (65), the fluctuations in the abundance of *Cx. pipiens* populations among different sites, are likely associated with the diversity of habitats and climatic/environmental conditions. Other factors, such as the presence/absence of suitable hosts (i.e., birds, horses, humans), not included in this study, may also impact mosquito abundance. Between 2019 and 2022, the yearly abundance of *Cx. pipiens* at each site has generally remained stable, showing a higher abundance along the coastal area (Figure 5). Fluctuations in mosquito abundance across different years could be attributed to variation in climatic factors (i.e., temperatures and rainfall). Additionally, small changes in the configuration of mosquito breeding habitats, induced by various atmospheric events, i.e., a shift in a riverbed course due to heavy precipitations, may also contribute to these fluctuations. The seasonal pattern of mosquito abundance revealed an increase during the spring months, reaching its peak in late summer, and subsequently declining in the autumn months. This population trend aligns with the typical seasonal pattern observed in the Mediterranean Basin, influenced by temperatures and photoperiod, which concurrently impact adult mosquito activity (14, 21, 27, 66).

Even though *Cx. pipiens* is widely distributed at our latitudes, its life cycle and the population seasonality are primarily influenced by local environmental conditions. The presence of local water pools is essential for the egg stage, and nearby suitable landscape features play a crucial role in supporting the adult stage, facilitating breeding, resting and survival (58). To fully capture these environmental prerequisites, our study employed two levels of detail in mapping areas suitable for *Cx. pipiens*: firstly, a fine-scale analysis of environmental conditions, as it accurately differentiates and map the mosquito-required environments for each life stage (67). Secondly, a broader-scale analysis of landscape variability near the sites was investigated, as it provides insights into the patchy territory favouring the mosquito proliferation (58, 68, 69). This study utilized a spatial resolution of 20 metres as reference, aiming to achieve a detailed representation of the landscape surrounding the entomological sites. Pixels of 20 × 20 meters were used to finely discretize the area around the sites, allowing for accurate identification of key landscape features such as presence of water, build-up areas, vegetation, grassland, etc., essential for sustaining the vector’s life stages. The satellite images subject to deep analyses were composed of 224 × 224 of those pixels, encompassing an area of 4480 × 4480 meters around each trap site, including its biodiversity composition and configuration. Each pixel in the satellite images represented the spectral reflectance for a specific date and wavelength acquisition.

The deep convolutional neural network models were provided with information on radiance wavelengths measured from satellites, without explicit land use or land cover classification, nor were pre-calculated indices, like NDVI, employed. The rationale behind this approach was to allow the algorithms to autonomously discern any relevant correlations, thereby surpassing any potential constraints of our existing knowledge. The Sentinel-2 spectral bands provide a comprehensive “picture” of the environment around the site, spanning a broader optical range than our traditional knowledge, from visible (B02—blue, B03—green, B04 red) to near infrared (B05, B06, B07, B8A for vegetation red-edge, WVP-B09 for Water Vapor), and short-wave infrared (B11, B12 snow-ice-cloud discrimination) wavelength of the electromagnetic spectrum. The multitude of these bands represents a more comprehensive set of information than the bands traditionally considered in such studies, as NDVI, Moisture Index, or Water Index, which rely on B04 and B08, B8A and B11, B03 and B08, respectively. In addition, convolutional neural networks, effectively extract meaningful features from images using a sequence of convolutional kernels and hierarchical layers (39): this approach enables the learning of local features (a group of pixels depicting a characteristic in the image), thus capturing important information in part of the image; CNNs also capture global patterns across the image (39, 40). The combination of local and global feature representation is crucial for capturing fine-scale details and broader-scale spatial conditions, making it a key component in successfully classifying mosquito habitat.

Three scenarios were tested in this study: the baseline model which considers environmental conditions at the time of mosquito collection; the multitemporal model, which considers conditions history up to 2 months prior to collection; and the MAGAT model which considers the relationship with nearby geographical sites and temperatures. The multitemporal model demonstrated the best performance, highlighting the importance of considering the temporal evolution of environmental conditions. The Sentinel-2 bands inherently contain some information about temperature variation: temperatures were included in the MAGAT model to identify similar sites, but the multitemporal model still achieved better performance. This suggests that the information contained in the multitemporal sequence of Sentinel-2 bands encompassed also the variations typically associated with temperature. The high frequency of Sentinel-2 overpasses at our latitudes is beneficial, providing updated images of the territory every 5 days. This temporal resolution is sufficient to detect and monitor the evolution of the vegetation growth, the phenological stages of crops in cultivated fields, and the impact of rainfall on vegetation and soils. These patterns are crucial for characterising the environment most suitable to *Cx. pipiens* presence and for monitoring population trends across seasons, assisting in optimal trap placement. In addition, 5 days interval align well with the temporal resolution of field activities. A finer resolution (less than 5 days) might offer more information, but this would approach the level of predicting daily mosquito population dynamics, which is beyond our study’s scope. The frequency of Sentinel-2 helps mitigate issues with cloudiness, a major drawback of optical measurements that rely on sunlight to collect information about the Earth’s surface. Frequent acquisitions increase the probability to have clear, cloud-free images.

The models were also tested against various time lags, meaning we considered eco-climatic conditions occurring a number of days before the collection. This “empty time” is valuable when the model is used as a base for operational predictive tools in support of surveillance activities. In both the baseline and MAGAT models, higher performances were reached by shorter temporal lags, indicating that environmental conditions closer to the collection time were the best predictors of mosquito presence/absence. On the other hand, the multitemporal model showed that the environmental conditions related to mosquito presence/absence were those occurring during the 2 months before the collection date, going back to 4 months (lag of 50 days): when a wider period of conditions were taken into account, the performance improved as the model “learned” the pattern of environmental conditions favouring the presence/absence of mosquito.

The multitemporal model utilized a sequence of 10 Sentinel-2 acquisitions: the long-term pattern identified with these 10 images provided the model with rich spectral information able to identify environmental changes that influence the presence/absence of *Cx. pipiens*. A similar result was obtained by Vincenzi et al. (42) for another mosquito species (*Culicoides imicola*) in Italy. Besides environmental data from multispectral images, the MAGAT model, which employs a graph architecture, considered also temperature and closeness to other sites to predict mosquito presence. However, its performances were closer to baseline metrics. The multitemporal model demonstrated high performances, capable of identifying the seasonal pattern of *Cx. pipiens* population: this result suggests that the added value of multitemporal analysis is greater than that of incorporating graphs and other predictors.

The use of Sentinel-2 data proposed in this study offers also the advantage that Copernicus represents a recently launched constellation of satellites, with missions planned to extend over the next several years. The Copernicus Sentinel-2 data, which comprise a substantial volume of information, holds significant potential for exploitation through deep learning techniques. On the other hand, the deep learning modeling adopted allows to replicate the models in other and wider geographical areas (with the same range of environmental conditions), for other vectors (relying on Sentinel-2 information), and for specific time-steps (at the beginning of the season) only, incorporating them into an operational tool.

Still, some drawbacks have to be highlighted: deep learning models require a huge amount of data for training and testing, which may not always be feasible when ground-truth data are of entomological nature, requiring in-field efforts for collection. However, this issue was considered in this study through the exploitation of pre-training strategies. Another critical issue is the requirement for high powerful supercomputers, along with the management of Sentinel-2 data, which demands advanced software and hardware resources (download, heavy files, etc.) and processing time. Additionally, the biological interpretation of the model presents another challenge: while the models can deeply understand the problem though neurons and layers and accurately predict classifications, the underlying biological relationships are not explicitly revealed. The interpretation of feature layers is not straightforward, so the deep learning methodologies could result as “black-box” outputs. This hampers the reliance on this kind of approaches from a biological point of view (70). Addressing the challenges of interpretability remains an active area of research, with advancements being crucial for the broader adoption of deep learning models in real-world applications. Overcoming these challenges required a multidisciplinary team effort in our research, bringing together different skills so to address issues like collecting ground truth data, processing biological samples, handling large amounts of data, and performing complex computations.

The findings of our study offer concrete support for the conceptualization and deployment of locally tailored entomological interventions, providing useful information for optimizing surveillance activities in the following seasons, enabling precise timing and location for trapping activities. This could be useful to support prevention and reduction of diseases transmitted by *Cx. pipiens*. The positive outcomes of our research pave the way for future actions, which include refining models with additional years of field data, integrating various surveillance data to improve generalization, extending the infrastructure to other diseases within the veterinary domain, investigating high-level biological features, and translating findings into an operational predictive tool for surveillance activities.

## 5 Conclusion

The need to optimise human and economic resources in conducting surveillance activities for vector-borne diseases necessitates efforts to identify areas at risk both spatially and temporally. The widespread availability of Earth Observation data related to vectors and diseases, coupled with the growing ability to apply sophisticated analysis and algorithms, enables the development of models and tools that facilitate and optimise the identification of such areas. This approach is based on the integration of diverse disciplines, expertise and data, leading to the achievement of the expected results. Specifically, in this research, the use of data from the Copernicus program at 20 m spatial resolution, along with deep learning models, have made it possible to solve a classification task in which entomological surveillance collections are predicted as positive or negative for *Cx. pipiens*, the main vector in Italy for West Nile virus, and the prediction is made with 15 days in advance. The model’s performances were satisfactory (F1 score was higher than 75.8% for any temporal lag in the baseline model; F1 score reached 81.4% in the multitemporal model and 80.9% in the MAGAT); results derived from this study will advance our ability to identify suitable times and areas for *Cx. pipiens* presence and high-risk exposure to VBDs within Italian landscapes.

The methodology employed here can be expanded to the national territory and to other vectors, supporting the Ministry of Health in developing strategies for the surveillance and control of the vectors and the diseases they transmit.

## Data availability statement

The data analyzed in this study is subject to the following licenses/restrictions: entomological data in Italy are not publicly available due to legal and security concerns. Requests to access these datasets should be directed to m.goffredo@izs.it.

## Ethics statement

The manuscript presents research on animals that do not require ethical approval for their study.

## Author contributions

CI: Visualization, Software, Methodology, Data curation, Conceptualization, Writing – review & editing, Writing – original draft. LB: Visualization, Software, Methodology, Formal analysis, Writing – review & editing, Writing – original draft. MdeA: Resources, Data curation, Writing – review & editing, Writing – original draft. ST: Resources, Data curation, Visualization, Software, Formal analysis, Writing – review & editing, Writing – original draft. AdiL: Resources, Software, Formal analysis, Data curation, Writing – review & editing, Writing – original draft. Sd’A: Resources, Data curation, Writing – review & editing, Writing – original draft. AP: Software, Investigation, Formal analysis, Methodology, Writing – review & editing. AB: Visualization, Writing – review & editing. DC: Software, Data curation, Writing – review & editing. MG: Validation, Resources, Funding acquisition, Conceptualization, Writing – review & editing. SC: Methodology, Conceptualization, Writing – review & editing. AC: Validation, Methodology, Funding acquisition, Conceptualization, Writing – review & editing, Writing – original draft.
